# Study of Tumor Growth under Hyperthermia Condition

**DOI:** 10.1155/2012/198145

**Published:** 2012-09-03

**Authors:** Qing Zhu, Aili Zhang, Ping Liu, Lisa X. Xu

**Affiliations:** ^1^School of Biomedical Engineering, Shanghai Jiao Tong University, Shanghai 200240, China; ^2^Med-X Research Institute, Shanghai Jiao Tong University, Shanghai 200240, China

## Abstract

The new concept of keeping primary tumor under control *in situ* to suppress distant foci sheds light on the treatment of metastatic tumor. Hyperthermia is considered as one of the means for controlling tumor growth. To simulate the tumor growth, a continuum mathematical model has been introduced. The newest understanding of the Warburg effect on the cellular metabolism and diffusion of the nutrients in the tissue has been taken into consideration. The numerical results are compared with the *in vivo* experimental data by fitting the tumor cell doubling time/tumor cell growth rate under different thermal conditions. Both the tumor growth curve and corresponding average glucose concentration have been predicted. The numerical results have quantitatively illustrated the controlling effect on tumor growth under hyperthermia condition in the initial stage.

## 1. Introduction

Cancer is the second major cause of human death in the world, and its mortality rate is growing every year [1]. Treatments include surgery, radiotherapy, chemotherapy, and gene therapy. Thermal therapy has also been intended to locally destroy tumor cells or enhance the body defense against tumor cells. However, recurrent rate of malignant tumor is still high [2], and the efficacy of the existing therapeutic means is yet to be improved. A new concept has been proposed recently that the primary tumor suppresses distal foci [3, 4]. This sheds new light on tumor treatment. Keeping the primary tumor *in situ* but restricting its size might enable the host to impede the development of distal foci and progression of metastasis. 

 For tumor growth, there are three distinct stages: avascular, vascular, and metastatic/invade stage. Mathematical models have been developed to perform parametric studies on factors influencing tumor growth or to evaluate the outcome of tumor treatment modalities [5, 6]. Model-based numerical studies would enable one to extrapolate more spatial and temporal information from the experimental findings and to make predictions [7]. Laird [8] first found that the tumor growth data-fitted Gompertz function could be used to simulate the entire growth curve, which was defined as an empirical model. Hu and Ruan [9] studied the suppression effect of immunity system on tumor growth by merging the Gompertz function into a cellular automaton model. Other mathematical models based on certain biological assumptions have also been attempted to predict tumor growth curve using fundamental physics, such as mass/energy conservation. Greenspan [10] introduced surface tension into the diffusion model developed by Burton [11]. Tumor growth/inhibition factors [12, 13], cell adhesions [14, 15], angiogenesis [16, 17] and invasion [18, 19] were further considered to describe tumor growth at different stages.

Models focusing on the avascular stage [20–27] have been well studied and could be easily applied to *in vitro* experiment. Ward and King [23, 24] and Casciari et al. [28] proposed a continuum mathematical model focusing on how nutrients' concentration affects tumor growth. These models typically consist of reaction-diffusion equations. Forbes [29] further incorporated energy metabolism (ATP production rate) into the growth model. However, most of these models have not taken the Warburg effect into consideration, which fundamentally differentiates the tumor cell metabolism from that of the normal cells.

In 1930, Warburg (1930) proposed that tumor cells preferentially underwent glycolysis when consuming glucose even under aerobic conditions. Unregulated glucose uptake and lactic acid production have been found in tumor cells as compared to normal cells [30, 31]. It indicates that tumor cells obtain energy to maintain their viability primarily relying on anaerobic metabolism. This phenomenon was termed as “the Warburg effect.” Anaerobic glycolysis consumes one molecule of glucose to produce 2 molecules of ATP as compared with oxidative phosphorylation which can produce 38 molecules of ATP [31–40]. Although the latter is much more efficient in glucose utilization, the rate of anaerobic glycolysis is much faster than aerobic metabolism. Therefore, the inefficient metabolism pathway might still supply enough energy for tumor cells to maintain their activities and differentiate at the cost of unreasonable consumption of glucose. The mechanisms causing the Warburg effect have been explained by gene mutation [38], signaling pathway alternations, possible defects in mitochondria [36, 41], and microenvironment deterioration (hypoxia or fluctuation of oxygen) [34, 37, 42]. Heiden et al. [32] have reported that biomass synthesis in tumor cells plays a role in the Warburg effect. Furthermore, he has determined nutrition utilizations in tumor cells: 85% of glucose converting to lactate in cytoplasm, 5% reacting in mitochondria, and 10% synthesizing biomass. As the metabolic activities greatly influence the growth of tumor, it is necessary to include this unique metabolic mode of tumor in mathematical models.

Although thermal treatment has been applied in clinical applications for many years, most of them were used as short-term treatments. There are three classes of the treatment strategies [43–45]: with a mild temperature at 40~41°C for 6~72 hours until the thermal dose is equivalent to 5 minutes at 43°C; a moderate temperature at 42~45°C for 15~60 minutes; and high temperature >50°C for 4~6 minutes. Both mild and moderate temperature treatments (40~45°C) termed as hyperthermia treatment impair mammalian cells by protein denaturation and membrane damage and could cause cell death *in situ* [46].

In the present study, a mathematical model of tumor growth has been built by combining ATP production rate and the mechanism of the Warburg effect. It is validated by the tumor growth measurements *in situ *and further applied to study the hyperthermia effect on tumor metabolism over a period of time. 

## 2. Theory

### 2.1. Experimental Study of Tumor Growth

 Animal study of tumor metabolisms under long-term hyperthermia has been studied [47]. In the study, tumor cells were injected into the back of Balb/c mice around 6~8 weeks. Mice were grouped randomly into the control and treatment groups with 6 in each group. In the treatment group, the tumor region of the mice was heated via a 15 mm-diameter circular heating pad for 28 days. On each day, it was heated for 12 hours with an interval of 12 hours. The supplying power of the heating pad was controlled to maintain the tumor surface tissue temperature at 39°C. Tumor sizes of both the control and treatment groups were measured and recorded at 3, 7, 10, 14, 21, 28 days, respectively. The experimental results are shown in Figures 2–5 with the permission of the authors.

### 2.2. Model Development

**Figure 1:**
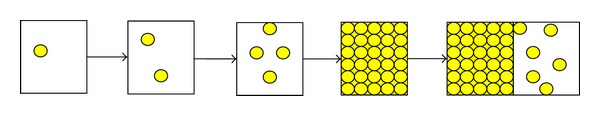
Schematic of tumor growth. The tumor starts with one single cell (represented by the yellow circle), and the cells differentiate continuously. When the infinitesimal unit of the space volume is filled up with the tumor cells, they grow into the next volume unit.

 At the avascular stage of a solid tumor, tumor cells proliferate and tumor grows like a spheroid without restriction. In our model, the tumor is assumed to start from one single tumor cell (as illustrated in Figure 1), and to grow into a homogenous spheroid over a period of time. This is an assumption used by many models [7, 10, 14, 16, 23, 24, 29] and acceptable when the diameter of tumor is less than 2~4 mm prior to microvascular development. In addition, only living tumor cells are supposed to take the space of the spheroid. The proliferation of tumor cells is related to local concentration of nutrients. In this model, nutrients are simplified to the main source of energy (glucose) only. Glucose diffuses passively into tumor tissue from the outer rim of the tumor, where its concentration remains at a constant over the initial tumor growth period.

Usually, the glucose is metabolized through two different pathways: aerobic and anaerobic, depending on the cell status and physiological conditions. According to the recent understanding of the Warburg effect [32, 38], in tumor metabolism, about 5% of glucose undergoes aerobic pathway, and 85% takes the anaerobic pathway to produce ATP. The rest of 10% glucose is utilized for biomass synthesis necessary for cell divisions.

Through anaerobic pathway, glucose first degrades into pyruvate, and pyruvate converts into lactate by lactate dehydrogenase in the cytoplasm. While in the aerobic metabolism, pyruvate will further react with oxygen, and produce water and carbon dioxide inside mitochondria. It is clear that not only the metabolic site and end products are different, but also the amount of energy produced differs. Through aerobic metabolism, one mole of glucose consumes 6 moles of oxygen and produces 38 moles of ATP, while in anaerobic metabolism only 2 moles of ATP per mole glucose could be produced without oxygen. These reactions are simplified and presented by the following formulae

Aerobic respiration:

(1) $$\begin{array}{c} {\text{C}}_{6}{\text{H}}_{12}{\text{O}}_{6}+6{\text{O}}_{2}+38\text{ADP}\to 6\text{C}{\text{O}}_{2}+6{\text{H}}_{\text{2}}\text{O}+38\text{ATP} \end{array}$$

Anaerobic respiration:

(2) $$\begin{array}{c} {\text{C}}_{6}{\text{H}}_{12}{\text{O}}_{6}\to 2{\text{C}}_{3}{\text{H}}_{6}{\text{O}}_{3}+2\text{ATP} \end{array}$$

The proliferation rate of tumor cells is supposed to rely on the ATP production rate, which is determined by the metabolic reactions:

(3) $$\begin{array}{c} Q_{\text{ATP}}=38Q_{g,\text{AR}}+Q_{\text{lac}}, \end{array}$$

where *Q*_*g*,AR_ is the glucose assumption rate due to aerobic respiration, *Q*_lac_ is the lactate production rate.

Following the hypothesis of Heiden [32], the glucose consumption rate due to aerobic metabolism and the lactate production rate could be derived from the uptake rate of glucose by tumor cells:

(4) Qg,AR=5%Qg,Qlac=85%×2×Qg,

where *Q*_*g*_ is the cellular glucose uptake rate.

Then the ATP production rate *Q*_ATP_ could be calculated:

(5) $$\begin{array}{c} Q_{\text{ATP}}=5\%\times 38\times Q_{g}+85\%\times 2\times Q_{g}. \end{array}$$

The cellular uptake rate of glucose depends on both the extracellular level of glucose concentration and the ability of cell uptake. The reaction is assumed to be enzyme reaction and followed Michaelis-Menten kinetics' equation [10, 25]:

(6) $$\begin{array}{c} Q_{g}=Q_{g\max}\frac{c_{g}}{c_{g}+K_{g}}, \end{array}$$

where *Q*_gmax_ is the maximum glucose uptake rate per tumor cell, *c*_*g*_ is the glucose concentration in tumor tissue, and *K*_*g*_ is saturate concentration.

 The concentrations of nutrients (both glucose and lactate) are assumed to be functions of tumor spheroid radius that are changing with the rate of cell proliferation [7]:

(7) $$\begin{array}{c} \frac{dc_{g}}{dt}=D_{g}\nabla^{2}c_{g}-Q_{g}l, \end{array}$$

where *D*_*g*_ is the diffusion coefficient of glucose and reported to be 1.05 ∗ 10^−6^ cm^2^/s for EMT6/R0 spheroid, and *l* is the number of living cell per unit volume.

The concentration of glucose at the boundary is decided by the average value of glucose concentration in blood—5 mM, and the tumor is assumed symmetric, thus the boundary conditions for (5) are as follows

Boundary condition:

(8) $$\begin{array}{c} {\frac{\partial c_{g}}{\partial t}|}_{r=0}=0,\;\;{c_{g}|}_{r=R}=5\;\text{mM}, \end{array}$$

where *R* is the radius of solid tumor.

 The distribution of lactate in tumor is determined by

(9) $$\begin{array}{c} \frac{dc_{\text{lac}}}{dt}=D_{\text{lac}}\nabla^{2}c_{\text{lac}}-Q_{\text{lac}}l, \end{array}$$

where *c*_lac_ is the concentration of lactate in tumor tissue, and *D*_lac_ is the effective diffusion coefficient of glucose and lactate. The boundary conditions are:

(10) $$\begin{array}{c} {\frac{\partial c_{\text{lac}}}{\partial t}|}_{r=0}=0,\;\;{c_{\text{lac}}|}_{r=R}=0\;\text{mM}. \end{array}$$

The diffusion coefficient of lactate could be determined from the glucose diffusivity [25]:

(11) $$\begin{array}{c} D_{\text{lac}}=D_{g}{\left(\frac{MW_{g}}{MW_{\text{lac}}}\right)}^{3/4}, \end{array}$$

where *MW*_*g*_ and *MW*_lac_ are molecular weights of glucose and lactate, respectively.

 Assuming that all viable space in tumor tissue is filled with living and tightly packed tumor cells (see Figure 1), the number of living cells per unit tumor tissue volume is

(12) $$\begin{array}{c} l=\frac{1}{V_{L}}, \end{array}$$

where *V*_*L*_ is the volume of a living tumor cell. It is obtained through *in vitro* measurement. Diameters of more than 150 suspended 4T1 tumor cells are measured and averaged to get the volume of a single cell. Its average radius is 13.41 *μ*m with a standard deviation of 2.21 *μ*m. With this radius, the tumor cell volume was calculated and the result is listed in Table 1. 

By solving the above listed equations together, the ATP production rate and concentrations of glucose and lactate inside the tumor tissue could be obtained. The relationship between ATP production rate and cell growth rate is proposed and well parameterized by Forbes et al. [29]:

(13) $$\begin{array}{c} k_{l}=A\frac{Q_{\text{ATP}}}{Q_{\text{ATP}}+K_{\text{ATP}}}, \end{array}$$

where *k*_*l*_ is cell growth rate of tumor cells, *A* is the maximum cell growth rate, and *K*_ATP_ is saturation ATP production rate.

In this model, tumor growth is considered as tumor cells moving outward while all space occupied. In other words, tumor growth is mainly due to living tumor cells' proliferation, then the moving velocity of the tumor spheroid rim is given as

(14) $$\begin{array}{c} v\left(t,R_{\left(t\right)}\right)=\frac{\int_{0}^{R_{\left(t\right)}}k_{l}lV_{L}r^{2}dr}{R_{\left(t\right)}^{2}}, \end{array}$$

where *r* is radial distant from center of tumor spheroid, and *R*_(*t*)_ is the radius of tumor spheroid at time *t*. The radius of tumor spheroid at time *t* + *dt* is

(15) $$\begin{array}{c} R_{\left(t+dt\right)}=\int_{t}^{t+dt}v\left(t,R_{\left(t\right)}\right)dt+R_{\left(t\right)}. \end{array}$$

The average concentration of the metabolites is calculated from the integration of the substances' distribution throughout tumor tissue divided by the tumor tissue volume as

(16) $$\begin{array}{c} {\overset{-}{c}}_{\left(t\right)}=\frac{\int_{0}^{R_{\left(t\right)}}\left(c\left(r\right)\cdot 4\pi r^{2}\right)dr}{\left(4/3\right)\pi R_{\left(t\right)}^{3}}. \end{array}$$

By substituting the dimensionless quantities and parameters as defined below into the above equations, the dimensionless equations could be obtained. 

The dimensionless variables are defined as

(17) $$\begin{array}{c} c_{g}^{*}=\frac{c_{g}}{c_{g,0}},\;\;c_{\text{lac}}^{*}=\frac{c_{\text{lac}}}{c_{g,0}},\;\;r^{*}=\frac{r}{R_{\left(t\right)}^{*}},\;\;t^{*}=\frac{t}{1/A}, \end{array}$$

where *c*_*g*,0_ = 5 mM.

Thus, the dimensionless equations are

(18) QATP∗=85%∗2∗Qg∗+5%∗38∗Qg∗Qg∗=QgQgmax⁡=cg∗cg∗+Kg/cg,0Qlac∗=QlacQgmax⁡=clac∗clac∗+Klac/cg,0dcg∗dt∗=1A(∇2cg∗−R(t)2Qgmax⁡DVLcg,0Qg∗)cg∗|r∗=1=1  dcg∗|r∗=0dt∗=0dclac∗dt∗=1A(∇2clac∗−R(t)2Qgmax⁡DVLcg,0Qlac∗)clac∗|r∗=1=0  dclac∗|r∗=0dt∗=0kl∗=klA=QATP∗QATP∗+KATP/Qgmax⁡R(t)=∫0t(v∗R(t)A)d(t∗1/A)+R0.

Tumor growth *in vivo* is actually a complex process, which involves many influencing factors such as gene mutation, immune system, tumor cell mechanism effect, tumor angiogenesis, metabolic waste, and tumor microenvironment. It is difficult to include all these factors into one single model. The present model is built based on energy production only and all other influencing factors are lumped into the maximum cell growth rate parameter *A*. The behavior of tumor growth is determined by the maximum cell growth rate *A* and it is determined by fitting to the animal experiment data. The other parameters used are all listed in Tables 1 and 2. The constants (*D*_*g*_, *Q*_*g*max⁡_) listed in Table 2 are taken from EMT6/Ro tumor, whose biophysical constants have been well studied. EMT6 is a mouse breast cancer cell line that grows in the Balb/C strain. 4T1 cells are assumed to be similar to EMT6 biophysically in the present modeling. 

To solve the equations listed above, discrete algorithm is built. The one-dimensional tumor space is divided into 1000 intervals evenly. The time increment Δ*t *is set to be small enough to guarantee the solver stability. The diffusion-reaction equations are then differentiated using the finite difference method. The tumor radius at certain time *t* is used as an input into the differentiated equation, and the forward elimination and backward substitution method is used to solve these differentiated equations to obtain the glucose concentration distribution, the corresponding ATP production rate, tumor growth rate, the velocity of the moving boundary of tumor, and the new tumor radius at a given time *t*. The updated tumor radius is then used as an input into the equations iteratively, until the difference between two iterations is small enough. For the next time step (*t* + Δ*t*), the radius of tumor is derived from the original radius at time *t* and radius increment during Δ*t*. This process is repeated until the final convergence reached in simulation.

The maximum cell growth rate parameter *A* is fitted from the animal experimental data. The reciprocal of *A* ranged from 0~50 h, with an interval of 0.2 h. Each *A* is then substituted to the mathematical model to obtain a simulated curve, which is compared with the animal experimental data using the coefficient of determination (R2) as a criteria. The optimal *A* is chosen in correspondance to the maximum achieved R2 value.

## 3. Results and Discussion

**Figure 2:**
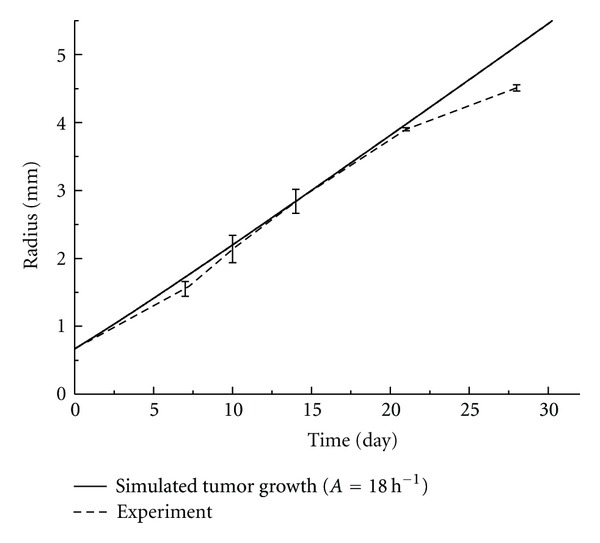
Tumor growth curve of the experimental measurements versus the numerical simulation of the control group.

**Figure 3:**
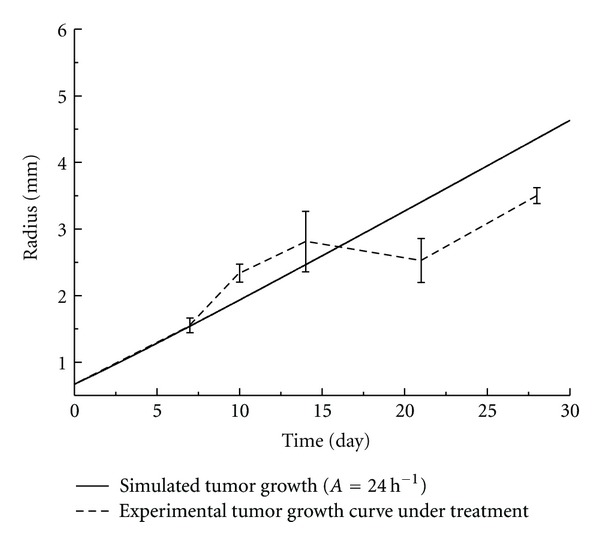
Tumor growth curve of the experimental measurements versus the numerical simulation of the treatment group (39°C) (*n* = 6).

 By fitting the numerical model with the experimentally measured tumor growth data, the parameter *A* for tumor growth with and without treatment was obtained. The fitted curve and the experimental results were shown in Figures 2 and 3, respectively.

 For the control group, the tumor growth curve during the first 28 days was well captured by the energy-based model developed in this study (Figure 2). The fitted maximum cell growth rate parameter *A* was (18 h)^−1^. However, after 28 days, the tumor growth slowed down and became nonlinear, which could no longer be described by the current mathematical model. It implied that alternations in tumor metabolism and some impedimental mechanisms might appear which led to cell death when tumor radius reached about 4~5 mm in diameter. These factors were not considered in the present model. 

Tumor growth in mice under long-term mild hyperthermia treatment was also fitted and shown in Figure 3. It was obvious that the growth rate of tumor was much slower and the fitted maximum cell growth rate *A* was (24 h)^−1^. The model predicted the general trend of tumor growth well in the first 15 days. The oscillation occurred in the growth curve that was not observed in the control group could be attributed to the thermal interference, although the causing mechanism is yet to be further explored. Moreover, it was clear that the model overestimated the tumor growth under the hyperthermia condition after 15 days. This indicates that more complex effects on the system might be triggered by hyperthermia after a period of time. It could be related to tumor angiogenesis, the activities of enzymes in different pathways associated with tumor cell metabolism and proliferation and so forth, which cannot be simply modeled based on energy consumption.

**Figure 4:**
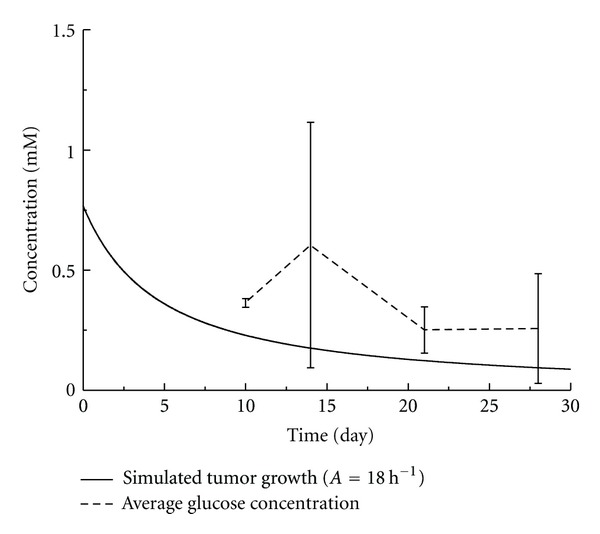
Average glucose concentration of the experimental measurements versus the numerical simulation of the control group.

**Figure 5:**
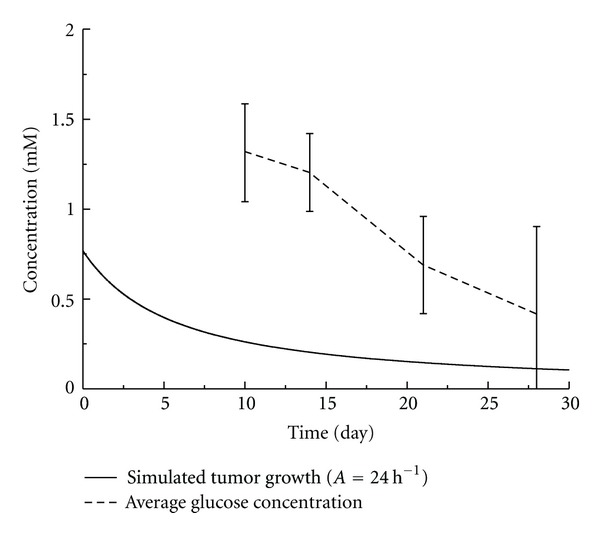
Average glucose concentration of the experimental measurement versus the numerical simulation of the treatment group.

With the fitted parameter *A*, the distribution of glucose in tumor has also been obtained. The average concentration of glucose in the tumor was calculated and compared to the experimental results. Seen from Figures 4 and 5, the average concentrations of glucose tended to decrease which agreed well with the experimental results. There existed significant differences between experimental and numerical results, especially for the group under the long-term mild hyperthermia treatment. This was likely because in the present model only glucose consumption for cell proliferation was considered. There could be excessive supply of glucose from the conceivable blood vessels inside tumor and the decreased cellular uptake of glucose due to heat. A more sophisticated model taking these factors into consideration should be developed to accurately predict the glucose distribution in tumor tissue.

**Figure 6:**
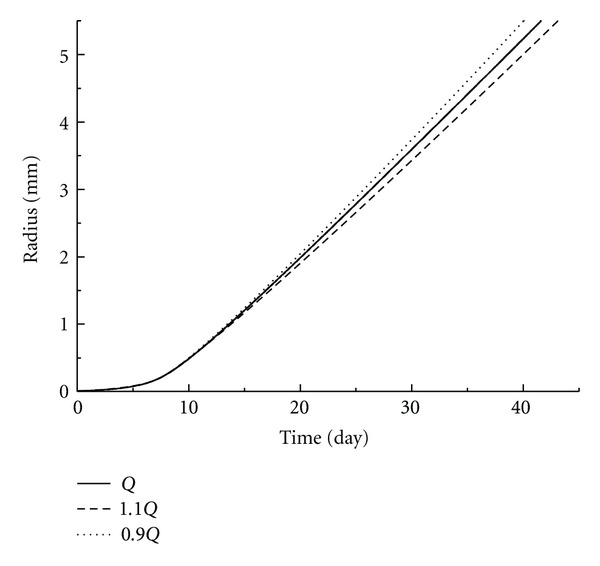
Parametric study of the tumor growth rate under different glucose uptake rates in tumor cells. Solid line stands for glucose uptake rate from reference [29]. Dot and dash lines represent 10% increase and decrease, respectively.

**Figure 7:**
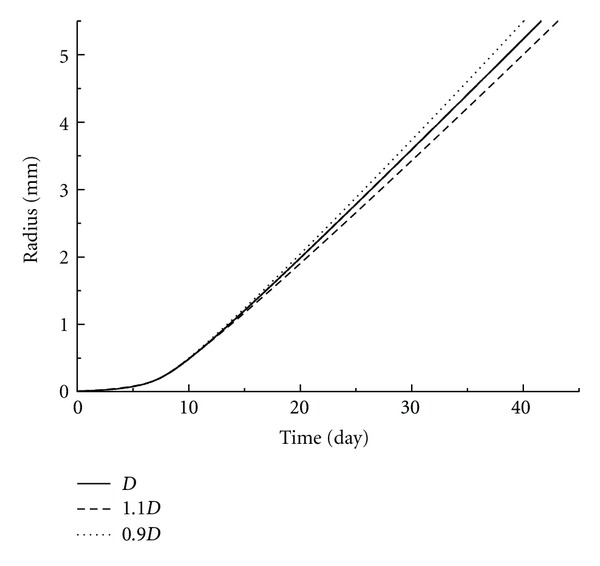
Parametric study of the tumor growth rate under different glucose diffusion coefficients in tumor tissue. Solid line stands for glucose diffusion value from reference [29]. Dot and dash lines represent 10% increase and decrease, respectively.

As there are no publications on the nutrition diffusion and cell uptake rate of 4T1 tumor cells, the data for EMT6/Ro tumor has been used. Although EMT6/Ro tumor cells are expected to possess similar properties, the dependence of these parameters on tumor species might also introduce some errors in the simulations. Therefore, parametric studies were performed and the influences on the glucose uptake rate and the diffusion coefficient were studied as shown in Figures 6 and 7. It was clear that the variation of tumor growth caused by 10% changes of either two factors was less than 5%.

In the present model, besides the Warburg effect, the influence of all other factors such as blood perfusion, immunity, and other growth or necrosis factors, has been lumped into a simple parameter *A*. Using tumor growth data from the experimental studies, the maximum tumor cell growth rate *in vivo* under the normal condition was fitted to be (18 h)^−1^, and the linear tumor growth at the early stage was successfully modeled without consideration of cell death. The growth was found to be excessively inhibited by the long-term mild hyperthermia treatment. In fact, during the treatment, both the temperature gradient and inhomogeneity existed, and the higher temperature could impair tumor cells by denaturation or destruction of cellular membrane, cellular skeleton, and nucleus [48]. The long-term mild hyperthermia treatment might also affect the activities of enzymes in different pathways associated with tumor cell metabolism and proliferation, stimulate the immunology factors such as hsp70, which could arouse body system defense and eliminate tumor cells specifically [49]. Besides, Song's study [50] revealed that under mild hyperthermia (41~42°C, 30 min) blood perfusion in tumor tissue could increase 1.5~2 folds as compared to that prior to the treatment. Tumor vasculature is very sensitive to heat, as it is loosely organized and usually lacks of smooth muscles [51–54]. Tumor cells would suffer from starvation due to the damage of the angiogenesis. All these influences on tumor growth are not linear and could not be accurately represented by a single parameter. This warrants further investigation into more detailed modeling of the long-term hyperthermia effect on tumor growth in the near future. 

## 4. Conclusion

An energy-based model linking tumor growth with cell proliferation rate has been developed in this study to investigate the hyperthermia treatment effect. In the model, the new understanding of the Warburg Effect was for the first time taken into account for tumor cellular metabolism regardless of the concentration of oxygen. The maximum cell growth rate was used as an integrated variable responding to changes under different environments. Trends of initial tumor growth and changes of the average glucose concentration in tumor were successfully modeled. The comparison of the maximum tumor cell growth rate has revealed a slowdown of tumor growth under the long-term mild hyperthermia condition. To accurately predict the tissue glucose level and the corresponding metabolites, especially under the long-term hyperthermia, the model needs to be further developed. 

## Figures and Tables

**Table 1:** List of variables used in modeling.

Variable	Description	Unit
*c*_*g*_	Glucose concentration	mM
*c*_lac_	Lactate concentration	mM
*Q*_*g*_	Glucose uptake rate	mol/cell/sec
*Q*_lac_	Lactate production rate	mol/cell/sec
*Q*_ATP_	ATP production rate	mol/cell/sec
*k*_*l*_	Cell proliferation time	1/h
*L*	Number of living cells in certain unit volume	cells/cm^3^
*R*	Radius of tumor spheroid	cm
*V*	Tumor growth velocity	cm/h
*T*	Growth time	h

**Table 2:** List of constants used in modeling (cell type—EMT6/Ro).

Variable	Description	Value	Authors
*D*_*g*_	Glucose diffusion coefficient	1.05 ∗ 10^−6^ cm^2^/s	Casciari et al., 1988 [28]
*Q*_*g*max⁡_	Maximum glucose uptake rate	1.33 ∗ 10^−16^ mol/(cell sec)	Casciari et al., 1992 [25]
*K*_*g*_	Glucose saturation constant	4.0 ∗ 10^−2 ^mM	Casciari et al., 1992 [25]
*V*_*L*_	Living cell volume	1.26 ∗ 10^−9^ cm^3^	Experiment
*c*_*g*,0_	Glucose concentration at tumor rim	5.5 mM or less	Freyer and Sutherland, 1986 [55]
*K*_ATP_	Cell growth saturation constant	3.75 ∗ 10^−19^ mol/cells	Venkatasubramanian et al., 2006 [29]
